# Engineering the Future of Stem Cells in Vascular Reconstruction: A Leap Towards Functional Endothelialized Tissue-Engineered Vascular Conduits

**DOI:** 10.1007/s12015-025-10968-8

**Published:** 2025-09-04

**Authors:** Haizam Oubari, Yanis Berkane, Maxime Jeljeli, Alexandre G. Lellouch, David M. Smadja

**Affiliations:** 1 Service de chirurgie plastique, reconstructrice et esthétique, Hospices Civils de Lyon, Lyon, France; 2 Suivi immunologique des thérapeutiques innovantes, UMR1236, Inserm, EFS, Université de Rennes, Rennes, France; 3 Service de chirurgie plastique, reconstructrice et esthétique, CHU de Rennes, Université de Rennes, Rennes, France; 4 Cedars-Sinai Medical Center, Los Angeles, CA USA; 5 Paris Cité University, INSERM UMR-S 970, Paris Cardiovascular Research Centre, Paris, France; 6 Hematology Department, Assistance Publique Hôpitaux de Paris-Centre Université de Paris (APHP-CUP), European Hospital Georges Pompidou, 56 rue Leblanc, Paris, 75015 France

**Keywords:** Tissue engineering, Vascular conduits, hiPSC-ECs, TEVCs, dHUAs, Decellularized human umbilical arteries, Shear stress, Bioreactor training

## Abstract

The transition from reconstructive to regenerative strategies in vascular surgery has intensified the need for grafts that are biocompatible, growth-capable, and resistant to thrombosis. Addressing this challenge, Park et al. introduce a groundbreaking method for engineering fully biological, endothelialized tissue-engineered vascular conduits (TEVCs) using decellularized human umbilical arteries (dHUAs) coated with human induced pluripotent stem cell-derived endothelial cells (hiPSC-ECs). These constructs undergo shear stress training in bioreactors, mimicking physiological blood flow to enhance endothelial functionality and anti-thrombotic properties. Upon implantation in animal models, the grafts showed long-term patency, resistance to thrombosis, and progressive replacement of hiPSC-ECs by host endothelial cells, highlighting their regenerative and integrative potential. The study emphasizes the pivotal role of hemodynamic conditioning and key regulators such as KLF2 in promoting endothelial quiescence and vascular homeostasis. It further explores alternative strategies like endothelial colony-forming cells (ECFCs) and microfluidic systems for flow-induced maturation. Clinically, this approach offers a promising, scalable avenue for patient-specific, immune-compatible vascular grafts applicable in congenital heart disease, dialysis access, vascular grafts and coronary bypass. While challenges such as long-term durability and mechanical reinforcement remain, this research marks a transformative step toward functional, off-the-shelf vascular grafts. Park et al.’s work bridges biomimicry with regenerative medicine, paving the way for next-generation vascular therapies rooted in endothelial mechanobiology and personalized bioengineering.

## Introduction

In the rapidly evolving landscape of transitioning from reconstructive to regenerative medicine, the quest for vascular grafts that seamlessly integrate with host tissues and mimic the properties of native vasculature remains a major focus [1]. Vascular tissue is subject to a wide range of biomechanical constraints [2], and achieving long-term graft patency remains a formidable challenge. Infection and thrombosis, in particular, remain critical hurdles to overcome, especially with synthetic biomaterials [3, 4]. Moreover, the lack of growth potential in commonly available prostheses and grafts significantly limits reconstructive options in the pediatric population, where the need for adaptable and durable vascular substitutes is critical [5]. The latest article from Park et al., recently published in *Cell Stem Cell* [6], presents an elegant and paradigm-shifting approach to the development of fully biologic endothelialized tissue-engineered vascular conduits (TEVCs), offering a promising path forward in vascular surgery but also in the treatment of congenital heart disease. Their work harnesses the power of human induced pluripotent stem cell-derived endothelial cells (hiPSC-ECs), leveraging shear stress training in flow bioreactors to enhance endothelial function. This strategy not only prevented thrombosis upon implantation but also promoted long-term vascular integration by enabling the gradual replacement of hiPSC-ECs with host-derived endothelial cells. The implications of these findings extend far beyond the laboratory, offering a clinically translatable solution for patients with vascular diseases.

Through this concise, comprehensive review, we wish to leverage their findings and discuss the current challenges and future perspectives of vascular tissue engineering.

## A Breakthrough in Vascular Tissue Engineering

Historically, vascular grafts have suffered from limitations such as thrombotic complications, immune rejection, and lack of growth potential, particularly when synthetic materials are used [7, 8]. Alternatively, previous attempts to engineer vascular conduits have utilized polymeric scaffolds seeded with autologous bone marrow cells. However, these strategies frequently resulted in graft stenosis, largely driven by inflammatory responses. Multiple challenges remain, including selecting materials with optimal porosity, mitigating inflammation, and ensuring long-term graft patency. Moreover, the need for highly tailored bioengineering solutions, coupled with their substantial expense and technical demands, continues to hinder broad clinical adoption. Moreover, the hemocompatibility of biomaterials -even those that are not inherently vascular but remain in direct contact with blood- has been a focus of research in recent years [9–11] with growing efforts aimed at transitioning from “bio-tolerated” materials to real biocompatibility [12]. In the same idea, Cohen et al. described a novel method for creating vascular chimerism by partially decellularizing donor organs, specifically removing only the vascular endothelium while preserving the rest of the tissue [13]. This partial decellularization reflects a deliberate balance between reducing immunogenicity and avoiding excessive matrix destruction, which can compromise organ function. By targeting only the vascular lining, the structural and biochemical integrity of the organ is largely maintained. Human placental endothelial progenitor cells (EPCs) were then used to repopulate the vasculature, effectively humanizing the graft’s inner lining [13]. The result is a chimeric organ with improved immune compatibility, a promising step for safer organ transplantation.

To address these constraints, the ultimate solution would be to develop off-the-shelf, pre-endothelialized universal scaffolds capable of promoting integration and maintaining long-term function. Another approach relies on post-implantation endothelialization of the construct, which, to date, represents one of the most promising strategies for achieving in vivo integration. As a relevant application, the safety of total artificial heart (TAH) implants, especially regarding thrombosis, has been associated with their potential for membrane re-endothelialization [14], with pericardial membranes playing a pivotal role. The concept of TAH has existed for over 50 years, originally developed as a temporary life-saving measure for patients with biventricular heart failure, primarily serving as a bridge to heart transplantation. A new generation of artificial hearts has emerged, including the Aeson TAH (A-TAH) developed by Carmat (Velizy-Villacoublay, France). The first human implantation of this device took place in 2013 in France [15], and more than 100 patients have been implanted so far. Unlike its predecessors, the A-TAH is designed to minimize hemolysis and prevent acquired von Willebrand syndrome, offering a more biocompatible solution [16]. It has been validated as a bridge to transplantation while requiring only minimal anticoagulation therapy [17]. Its unique design incorporates hemocompatible materials, with all blood-contacting surfaces covered by membranes derived from bovine pericardium, an engineered xenogeneic tissue widely used in bioprosthetic valves for over three decades [18]. The use of glutaraldehyde-fixed xenogeneic tissue in the A-TAH significantly reduces immunogenicity, making it the most advanced bioprosthetic artificial heart to date. Recent studies have suggested that this tissue may serve as a scaffold for neo-endothelialization, promoting the formation of a non-thrombogenic surface [19]. Emerging evidence indicates that endothelial cells responsible for this process originate from circulating blood [20], further supporting the notion that an optimal total artificial heart could integrate elements of both xenotransplantation and cutting-edge biomedical engineering.

These findings provide crucial clinical and physiological validation of the need to reendothelialize materials in direct contact with blood, reinforcing the paradigm shift in vascular biomaterial design. In this context, the groundbreaking study by Park et al. represents a revolutionary departure from traditional approaches, leveraging decellularized human umbilical arteries (dHUAs) as biological scaffolds [6]. By closely replicating the native extracellular matrix (ECM) of blood vessels, this approach paves the way for a new era in vascular engineering, bridging biomimicry and regenerative medicine to achieve superior hemocompatibility and long-term functionality. Their methodology involves coating the luminal surface of dHUAs with hiPSC-ECs under precisely controlled shear stress conditions in bioreactors, a process that induces endothelial maturation and antithrombotic functionality. This training is critical, as it mimics the physiological hemodynamic forces experienced by endothelial cells in vivo, thereby enhancing their resistance to thrombosis and optimizing their functional longevity. Upon implantation into the inferior vena cava (IVC) of nude rats, these endothelialized TEVCs remained patent, resisted thrombus formation, and were progressively repopulated by host endothelial cells, a critical hallmark of successful vascular graft integration.

## Shear Stress Training: A Game-Changer in Endothelial Function

**Fig. 1:**
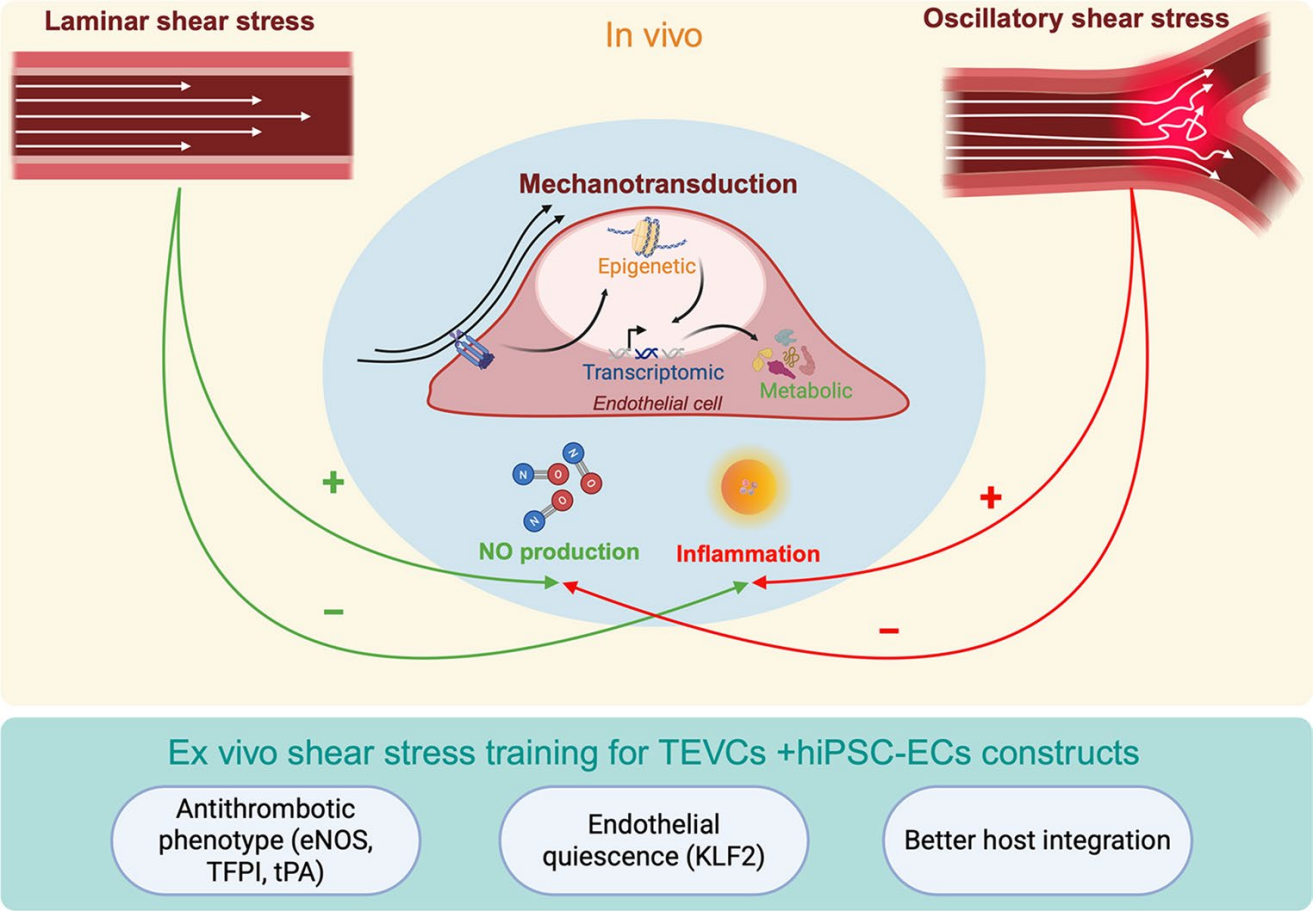
The impact of shear stress on endothelial cells and its utilization for biosconstruct preconditioning

Shear stress, the tangential force exerted by blood flow on the endothelium, is increasingly recognized as a critical regulator of vascular homeostasis, endothelial phenotype, and tissue-engineered graft performance. In physiological conditions, steady laminar shear stress (LSS), typically observed in straight arterial regions, plays a protective role by promoting endothelial quiescence, nitric oxide (NO) production, and anti-inflammatory gene expression. In contrast, the disturbed flow patterns found at vascular curves, branch points, and bifurcations, known as oscillatory shear stress, promote endothelial dysfunction by triggering inflammatory responses and activating gene expression profiles associated with atherosclerosis susceptibility (Fig. 1). The mechanotransduction of shear stress is mediated through a complex network of mechanosensors and signaling cascades that ultimately converge on cytoprotective gene expression and endothelial alignment along the flow vector​​. Furthermore, recent insights into metabolo-epigenetics have revealed that shear stress not only affects transcriptomic profiles but also reshapes the epigenetic and metabolic landscape of endothelial cells, suggesting durable reprogramming effects that extend beyond the immediate mechanical stimulus​​​ [21, 22]. Shear stress training emerges bioengineering tool that conditions stem cell-derived endothelial cells into a therapeutically competent state to promote endothelial quiescence and stability, reduce early thrombosis, and mitigate inflammation [23, 24].

One of the most compelling aspects of the Park’s study is the role of shear stress conditioning in the maturation of hiPSC-ECs. However, translating these principles into functional TEVCs has remained an ongoing challenge, which Park et al. addressed by implementing a gradual shear stress training regimen. The exposure of hiPSC-ECs to an initial arterial-like shear stress of 15 dynes/cm², followed by a ramp-down to 5 dynes/cm², enabled simulating the hemodynamic environment of the IVC. This process induced robust expression of endothelial nitric oxide synthase (eNOS), tissue factor pathway inhibitor (TFPI), and tissue plasminogen activator (tPA), key markers of an antithrombotic endothelial phenotype. In contrast, TEVCs seeded with static (non-trained) hiPSC-ECs exhibited higher fibrinogen adsorption, reduced endothelial coverage, and increased thrombus formation upon implantation. The importance of this finding cannot be overstated. It highlights the necessity of shear training in generating physiologically competent endothelial cells capable of maintaining vessel patency. This principle could be extended beyond vascular grafts to bioengineered organ vasculature, endothelialized stents, and even synthetic microvasculature for organ-on-chip models. Recent advancements in vascular tissue engineering have emphasized the critical role of endothelialization in improving graft function and longevity. Among these innovations, the study by Park et al. has demonstrated how endothelial shear stress conditioning enhances vascular graft performance by optimizing the functionality of hiPSC-ECs. Blood flow-induced shear stress plays a pivotal role in regulating endothelial cell function and vascular structure. The transcription factor KLF2 has been identified as a key mediator of this response, promoting an anti-inflammatory and quiescent endothelial phenotype under laminar flow conditions [25–29]. While its effects have been extensively studied in two-dimensional models, its application in three-dimensional engineered vasculature remains underexplored. To address this gap, researchers have developed microvascular networks (MVNs) incorporating KLF2-based endothelial cell flow sensors within microfluidic systems, applying continuous flow to mimic physiological conditions [25]. This approach has yielded significant findings, including increased vessel diameter, reduced vascular resistance, and enhanced barrier function, all of which contribute to reduced platelet adhesion and improved hemocompatibility. These results underscore the potential of KLF2-based flow training as a transformative method for optimizing bioengineered vascular grafts. The role of shear stress is further emphasized in vascular dysfunction in organ transplantation, highlighting the detrimental effects of the absence of intravascular flow on endothelial function [30]. Temporary flow cessation, such as observed during cold organ storage, leads to acute endothelial dysfunction and apoptosis due to the rapid loss of KLF2 expression and its downstream vasoprotective targets. This endothelial impairment is a key contributor to post-transplant graft failure through the noxious endothelial ischemia-reperfusion injury (IRI) cascade [31, 32]. Recent work has shown that supplementing preservation solutions with pharmacological agents, such as simvastatin, can restore KLF2 expression, maintaining endothelial integrity and preventing apoptosis [26, 27, 30]. These findings underscore the essential role of flow in sustaining endothelial homeostasis and reinforce the necessity of shear stress conditioning in bioengineered vascular grafts to enhance their longevity and function. Advancements in vascular tissue engineering have further explored KLF2-mediated endothelial conditioning through microfluidic-based systems. Such an approach involves engineered microvascular networks (MVNs) incorporating KLF2-based endothelial cell flow sensors within microfluidic devices, where continuous flow application mimics physiological conditions. This innovative system has demonstrated significant benefits, including increased vessel diameter, reduced vascular resistance, and improved endothelial barrier function, ultimately reducing platelet adhesion and thrombotic risks. These results confirm that shear stress is a biomechanical force and a critical determinant of endothelial health [33].

## Endothelial Replacement and Host Integration

For long-term maintenance of vascular permeability and growth potential, bioconstructs must achieve effective biointegration with the host. Fibrotic tissue has long been investigated as a potential source for autologous grafting, but despite initial biocompatibility, long-term outcomes remain [34, 35]. Notably, vessels possess their own microvascular and lymphatic networks, collectively known as the *vasa vasorum* [36]. These structures play an integral role in vascular physiology and repair mechanisms, as seen in conditions such as coronary artery disease and cardiac allograft vasculopathy [37], repair, such as in the case of coronary disease, and cardiac transplant arteriopathy [38]. These examples highlight the critical importance of enabling inosculation [39] and neovascularization of implanted constructs to ensure optimal biointegration [40]. This process may also be essential for facilitating re-endothelialization of the grafted vessel, a key element in ensuring both its short- and long-term function and viability. The principle of integration, both endothelial and transmural, is fundamental to the success of tissue-engineered vascular constructs. As such, the selection of scaffold material becomes a critical determinant of performance. This concept has been central to the field of tissue engineering since its inception over four decades ago. Synthetic scaffolds offer reliable mechanical strength and structural integrity, yet they often fall short in providing the biological cues necessary for host cell recruitment and integration. In contrast, biologically derived scaffolds, though more fragile mechanically, tend to promote superior biointegration and immunocompatibility. Hydrogels, too, have demonstrated potential by supporting cell viability and promoting matrix remodeling; however, they are often limited by their lack of mechanical robustness and cellular integration [41–43]. Emerging hybrid strategies seek to bridge this divide by combining synthetic materials with the biological complexity of natural extracellular matrices [44, 45]. Specifically, biologic scaffolds derived from donor tissues of either the same or different species, once decellularized, are emerging as valuable platforms. They retain natural structural features and biochemical cues, supporting both cell repopulation and the regeneration of vascular tissue [46].

Park et al. showed that the hiPSC-ECs were progressively replaced by host-derived endothelial cells within a matter of weeks post-implantation. By day 7, the majority of hiPSC-ECs had been replaced, yet key antithrombotic markers such as TFPI, tPA, and glycocalyx components remained intact, suggesting that host ECs had seamlessly assumed endothelial function. This phenomenon underscores the biocompatibility and regenerative potential of these TEVCs, as host cells integrate into the graft and contribute to long-term endothelial maintenance. Unlike synthetic grafts that may induce chronic inflammation or immune rejection, the use of biological scaffolds coupled with transient hiPSC-EC coating allows for a more natural transition to host-derived endothelium.

## Immunomodulatory Properties of Engineered Stem cell-derived Endothelial Cells

Recent advances in vascular tissue engineering have emphasized the central role of endothelial immunobiology in the development of functional and durable immune-compatible vascular grafts [47, 48]. ECs are immunologically active sentinels that orchestrate local and systemic immune responses [49]. Under homeostatic conditions, quiescent ECs maintain vascular integrity and immune tolerance by expressing low levels of major histocompatibility complex (MHC) class I molecules and negligible levels of MHC class II or costimulatory molecules such as CD80 and CD86 [50]. They also secrete anti-inflammatory mediators such as transforming growth factor-beta (TGF-β) [51, 52] and nitric oxide (NO) [53], which preserves the endothelium balance [54]. However, under inflammatory or mechanical stress, ECs undergo phenotypic switching with an upregulation of MHC class II [55, 56], adhesion molecule ICAM-1 [57], and proinflammatory cytokines such as IL-6 and IL-1β [58, 59] thus which promotes the recruitment of monocytes and neutrophils and triggers the activation of CD4^+^ T cells [57].

This immunologic plasticity is particularly relevant in the context of engineered grafts, where ECs can influence both innate and adaptive immune responses at the blood-graft interface. Park et al. harnessed this immunoregulatory potential by exposing hiPSC-ECs to physiological shear stress in a flow bioreactor, inducing a quiescent, anti-inflammatory phenotype. These vascular constructs showed increased expression of antithrombotic markers (eNOS, KLF2, KLF4) and reduced levels of thrombogenic markers (E-selectin, P-selectin, ICAM-1, VCAM-1) compared to statically cultured EC-coated grafts [6]. Shear stress–responsive transcription factors such as Krüppel-like factor 2 (KLF2) and KLF4 are central to this process, repressing NF-κB signaling [60] and increasing endothelial nitric-oxide synthase expression [61] which promotes vascular homeostasis [62]. Importantly, Park et al. also observed gradual replacement of the implanted hiPSC-ECs by host-derived ECs, suggesting a process of immune-mediated remodeling and integration, rather than immune rejection. This phenomenon highlights the possibilities for the temporary use of engineered ECs as immunomodulatory scaffolds, priming the local environment for host repopulation. However, the immunogenicity of hiPSC-derived cells remains a significant challenge. Although hiPSC-ECs are generally considered immune-compatible when derived from autologous sources [63], studies have shown variable expression of danger-associated molecular patterns (DAMPs) such as High mobility group box 1 (HMGB1) [64, 65] and HLA molecules, particularly when cells are incompletely differentiated or exposed to inflammatory stimuli [66]. Consequently, allogeneic hiPSC-ECs used in off-the-shelf constructs must be carefully evaluated for their potential to activate host T cells or NK cells. Significant advances have been recently made to overcome the immunogenicity of allogeneic cell therapies, including strategies such as HLA matching, gene editing, and immunosuppressive coatings. Remarkably, research by Schrepfer and colleagues showed that endothelial cells generated from mouse or human induced pluripotent stem cells (iPSCs), modified to lack MHC class I and II expression and to overexpress CD47, were able to persist long-term in allogeneic hosts with complete MHC mismatch. These engineered cells avoided immune system attack without requiring immunosuppressive therapy and maintained their ability to fulfill complete differentiation [67]. Overall, these findings highlight the importance of integrating immunological considerations into vascular graft design to develop universally compatible and long-lasting regenerative vascular therapies.

## Clinical Implications: From Bench To Bedside

**Fig. 2:**
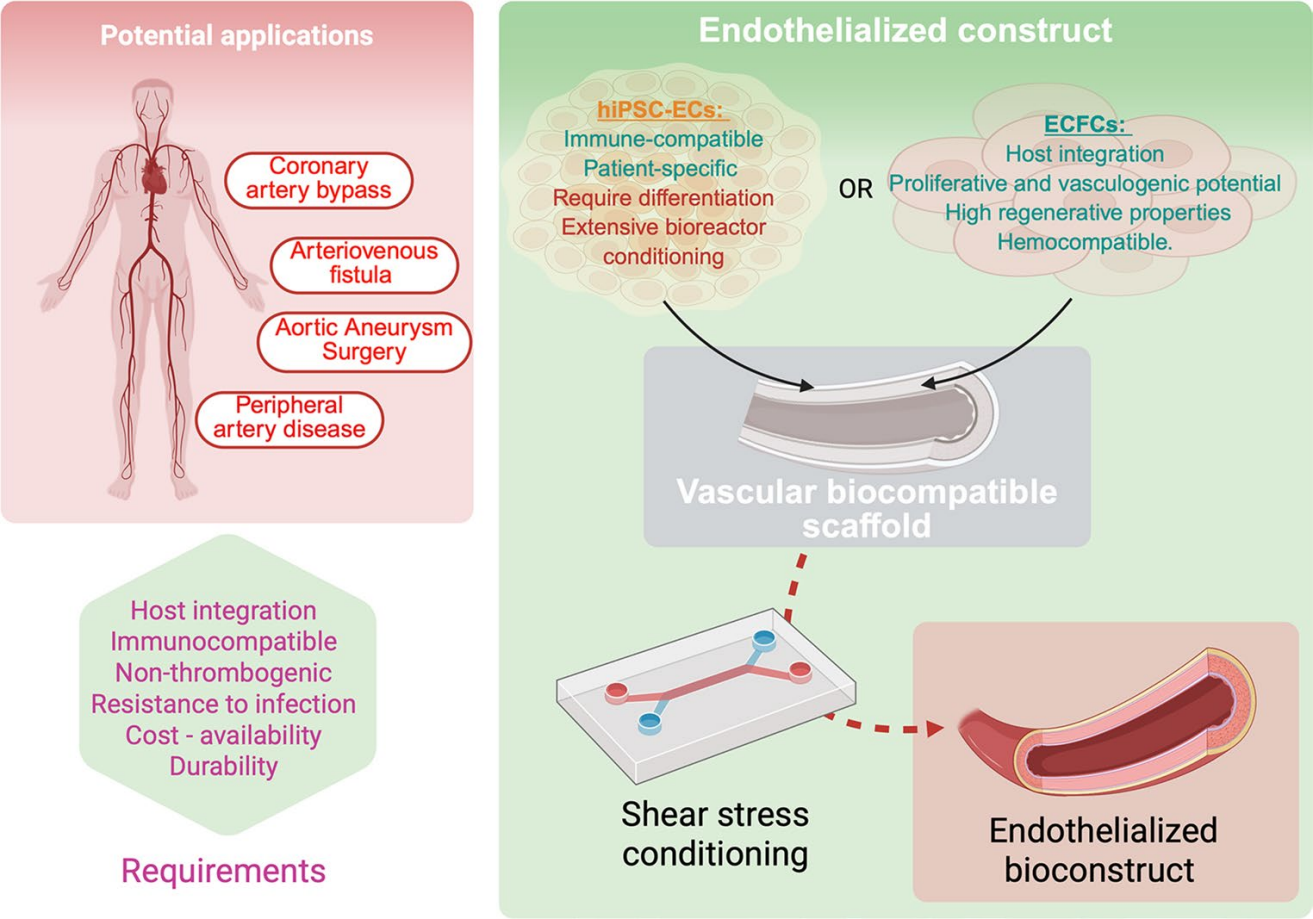
Clinical relevance and perspectives for endothelialized vascular bioconstructs

Cryopreserved arterial allografts harvested from human cadavers exhibit low immunogenicity due to cellular depletion during processing [68]. They are primarily used in vascular surgery when autologous grafts are unavailable, especially for infected prosthetic graft replacements or critical limb ischemia [69]. Despite their reduced antigenicity, chronic immune-mediated degradation may occur over time [70]. Allografts offer better resistance to infection compared to synthetic prostheses [71]. However, their long-term durability remains a clinical challenge [72]. Beyond congenital heart disease, endothelialized biomaterials hold significant potential for applications in peripheral artery disease, arteriovenous fistula creation for dialysis patients, and coronary artery bypass grafting (CABG). This mechanistic gap is also critical in tissue engineered approaches in reconstructive surgery [73]. The role of endothelialized constructs in personalized medicine is promising particularly for endothelial derived cells [74, 75], as patient-derived hiPSC-ECs could be used to generate immune-compatible, patient-specific vascular grafts and vascularized engineered constructs. In addition to hiPSC-ECs, endothelial colony-forming cells (ECFCs) emerge as a compelling alternative for this approach [48, 76–78]. ECFCs represent a promising source of endothelial cells due to their strong proliferative and vasculogenic potential as well as host integration and hemocompatibility properties. Unlike hiPSC-ECs, which require differentiation protocols and extensive bioreactor conditioning, ECFCs are naturally primed for vascular integration and have demonstrated high regenerative properties in preclinical models, particularly contrasting to cells embryonic stem cells or adult blood-derived cells [79]. Integrating ECFCs with shear stress-based endothelial conditioning, such as KLF2 activation [80, 81], could further enhance the stability, functionality, and long-term patency of engineered vascular grafts. This dual approach, leveraging the biomechanical influence of shear stress alongside the inherent vasculogenic properties of ECFCs, could pave the way for next-generation bioengineered vessels, offering improved hemocompatibility and enhanced graft integration in clinical applications (Fig. 2).

While Park’s study represents a major advance in vascular tissue engineering, several key challenges remain:

1. **Long-Term Functionality**: While short-term patency was demonstrated, extended studies are needed to evaluate TEVC durability over several months to years. However, results obtained from small-caliber vessels in rodent models cannot be directly extrapolated to clinical settings. The porcine model is considered superior, offering more relevant anatomical and physiological similarities to human vasculature for translational evaluation.
2. **Mechanical Strength** - Although dHUAs provide a natural ECM scaffold, further reinforcement may be required to withstand higher-pressure arterial environments.
3. **Clinical Translation** - Standardization of hiPSC-EC differentiation protocols and scalable bioreactor systems will be crucial for moving this technology from preclinical models to human trials. Combination with in vivo preconditioning approaches could improve biocompatibility and full vascular integration [82].
4. **Host-related limitations** - While scaffold properties, endothelialization, and hemodynamic factors are critical for long-term patency, clinical outcomes are also heavily influenced by patient- and vessel-related factors. The burden of cardiovascular risk factors, such as smoking, chronic low-grade inflammation, dyslipidemia, and diabetes, remains among the strongest determinants of long-term graft performance [83]. Moreover, health disparities and the high prevalence of cardiovascular disease may partly reflect genetic differences in endothelial shear stress responses, potentially influencing endothelialization and vascular integration. Indeed, Wei et al. demonstrated that ECFCs from African American and Caucasian American donors exhibit significant differences in gene expression, particularly within shear stress–responsive pathways—a key regulator of endothelial phenotype, vascular tone, and homeostasis [81]. These genetically influenced variations suggest that host-related factors, including ancestral genomic background, can modulate endothelial cell behavior under hemodynamic forces, potentially affecting processes such as endothelialization, vascular repair, and integration of grafts or devices. Hemodynamic conditions, particularly shear stress, are essential considerations; as observed with free grafts in CABG, conduits originating from low-pressure circulations may be especially vulnerable when exposed to high systemic arterial pressures [84, 85]. Additionally, the quality of the peripheral outflow could have a profound impact on durability. Arterial grafts anastomosed to diffusely diseased recipient arteries, common in advanced diabetes, coronary artery disease, or peripheral arterial disease, show significantly reduced longevity [86]. These limitations are not unique to decellularized scaffolds and must be acknowledged when evaluating any allogeneic vascular graft.

Additionally, the study raises intriguing questions about the mechanisms driving host endothelial cell recruitment. Future research should explore whether specific cytokine signals or extracellular vesicles mediate the transition from hiPSC-ECs to host-derived endothelium. Understanding these pathways could unlock new strategies for enhancing vascular graft integration. The concepts of bioengineering have progressively evolved, from initially attempting to mimic isolated biological properties to now encompassing the full spectrum of graft maturation, both ex vivo and in vivo, with an emphasis on early biocompatibility and long-term functionality.

All in all, Park et al. have provided a compelling blueprint for the next generation of vascular grafts, one that seamlessly integrates stem cell technology, endothelial biomechanics, and regenerative medicine. Their approach addresses many of the historical challenges associated with vascular tissue engineering, offering a clinically relevant, thrombosis-resistant, and host-integrating solution. As we stand at the intersection of bioengineering and translational medicine, studies like this push the boundaries of what is possible in cardiovascular surgery and vascular replacement therapies. With continued innovation, endothelialized biomaterials may soon become a mainstay in treating vascular diseases, paving the way for a new era of personalized, regenerative medicine. The journey from bench to bedside is never straightforward, but with such advancements, the future of vascular surgery looks undeniably promising.

## Data Availability

No datasets were generated or analysed during the current study.

## References

[1] Ding, X., Sha, D., Sun, K., & Fan, Y. (2025). Biomechanical insights into the development and optimization of small-diameter vascular grafts. *Acta Biomaterialia*, *S1742-7061*(25), 00270–00273. 10.1016/j.actbio.2025.04.028 10.1016/j.actbio.2025.04.028 40239752

[2] Scholpp, S., Hoffmann, L. A., Schätzlein, E., Gries, T., Emonts, C., & Blaeser, A. (2025). Interlacing biology and engineering: An introduction to textiles and their application in tissue engineering. *Materials Today Bio*, *31*, 101617. 10.1016/j.mtbio.2025.101617 40124339 10.1016/j.mtbio.2025.101617 PMC11926717

[3] Giannico, S., Hammad, F., Amodeo, A., Michielon, G., Drago, F., Turchetta, A., Sanders, S. P., et al. (2006). Clinical outcome of 193 extracardiac Fontan patients: The first 15 years. *Journal of the American College of Cardiology,**47*(10), 2065–2073. 10.1016/j.jacc.2005.12.065 16697327 10.1016/j.jacc.2005.12.065

[4] Spadaccio, C., Rainer, A., Barbato, R., Trombetta, M., Chello, M., & Meyns, B. (2019). The long-term follow-up of large-diameter Dacron^®^ vascular grafts in surgical practice: A review. *The Journal of Cardiovascular Surgery*, *60*(4), 501–513. 10.23736/S0021-9509.16.08061-7 24727799 10.23736/S0021-9509.16.08061-7

[5] Blum, K. M., Mirhaidari, G. J. M., & Breuer, C. K. (2022). Tissue engineering: Relevance to neonatal congenital heart disease. *Seminars in Fetal & Neonatal Medicine*, *27*(1), 101225. 10.1016/j.siny.2021.101225 33674254 10.1016/j.siny.2021.101225 PMC8390581

[6] Park, J., Riaz, M., Qin, L., Zhang, W., Batty, L., Fooladi, S., & Qyang, Y. (2025). Fully biologic endothelialized-tissue-engineered vascular conduits provide antithrombotic function and graft patency. *Cell Stem Cell*, *32*(1), 137–143e6. 10.1016/j.stem.2024.11.006 39644899 10.1016/j.stem.2024.11.006 PMC11698629

[7] Attard, C., Huang, J., Monagle, P., & Ignjatovic, V. (2018). Pathophysiology of thrombosis and anticoagulation post Fontan surgery. *Thrombosis Research*, *172*, 204–213. 10.1016/j.thromres.2018.04.011 29685556 10.1016/j.thromres.2018.04.011

[8] Wilson, W. R., Bower, T. C., Creager, M. A., Amin-Hanjani, S., O’Gara, P. T., Lockhart, P. B., & American Heart Association Committee on Rheumatic Fever, Endocarditis, and Kawasaki Disease of the Council on Cardiovascular Disease in the Young; Council on Cardiovascular and Stroke Nursing; Council on Cardiovascular Radiology and Intervention; Council on Cardiovascular Surgery and Anesthesia; Council on Peripheral Vascular Disease; and Stroke Council. (2016). Vascular graft infections, mycotic aneurysms, and endovascular infections: A scientific statement from the American heart association. *Circulation*, *134*(20), e412–e460. 10.1161/CIR.0000000000000457 27737955 10.1161/CIR.0000000000000457

[9] Stratton, J. R., Thiele, B. L., & Ritchie, J. L. (1983). Natural history of platelet deposition on Dacron aortic bifurcation grafts in the first year after implantation. *The American Journal of Cardiology*, *52*(3), 371–374. 10.1016/0002-9149(83)90141-8 6223522 10.1016/0002-9149(83)90141-8

[10] Nascimbene, A., Bark, D., & Smadja, D. M. (2024). Hemocompatibility and biophysical interface of left ventricular assist devices and total artificial hearts. *Blood*, *143*(8), 661–672. 10.1182/blood.2022018096 37890145 10.1182/blood.2022018096 PMC10900168

[11] Wissing, T. B., Bonito, V., Bouten, C. V. C., & Smits, A. I. P. M. (2017). Biomaterial-driven in situ cardiovascular tissue engineering-a multi-disciplinary perspective. *NPJ Regenerative Medicine*, *2*, 18. 10.1038/s41536-017-0023-2 29302354 10.1038/s41536-017-0023-2 PMC5677971

[12] Ratner, B. (2023). Vascular grafts: Technology success/technology failure. *BME Frontiers*, *4*, 0003. 10.34133/bmef.0003 37849668 10.34133/bmef.0003 PMC10521696

[13] Cohen, S., Partouche, S., Gurevich, M., Tennak, V., Mezhybovsky, V., Azarov, D., & Nesher, E. (2021). Generation of vascular chimerism within donor organs. *Scientific Reports*, *11*(1), 13437. 10.1038/s41598-021-92823-7 34183759 10.1038/s41598-021-92823-7 PMC8238957

[14] Golden, M. A., Hanson, S. R., Kirkman, T. R., Schneider, P. A., & Clowes, A. W. (1990). Healing of polytetrafluoroethylene arterial grafts is influenced by graft porosity. *Journal of Vascular Surgery,**11*(6), 838–844. 10.1067/mva.1990.18047. discussion 845. 2359196 10.1067/mva.1990.18047

[15] Latrémouille, C., Carpentier, A., Leprince, P., Roussel, J.-C., Cholley, B., Boissier, E., Smadja, D. M., et al. (2018). A bioprosthetic total artificial heart for end-stage heart failure: Results from a pilot study. *The Journal of Heart and Lung Transplantation: The Official Publication of the International Society for Heart Transplantation,**37*(1), 33–37. 10.1016/j.healun.2017.09.002 28986001 10.1016/j.healun.2017.09.002

[16] Poitier, B., Chocron, R., Peronino, C., Philippe, A., Pya, Y., Rivet, N., M Smadja, D., et al. (2022). Bioprosthetic total artificial heart in autoregulated mode is biologically hemocompatible: Insights for multimers of von Willebrand factor. *Arteriosclerosis Thrombosis and Vascular Biology,**42*(4), 470–480. 10.1161/ATVBAHA.121.316833 35139659 10.1161/ATVBAHA.121.316833

[17] Smadja, D. M., Ivak, P., Pya, Y., Latremouille, C., Gustafsson, F., Roussel, J. C., Netuka, I., et al. (2022). Intermediate-dose prophylactic anticoagulation with low molecular weight heparin is safe after bioprosthetic artificial heart implantation. *The Journal of Heart and Lung Transplantation: The Official Publication of the International Society for Heart Transplantation,**S1053–2498*(22), 01956–8. 10.1016/j.healun.2022.05.017 10.1016/j.healun.2022.05.017 35715318

[18] Neuenschwander, S., & Hoerstrup, S. P. (2004). Heart valve tissue engineering. *Transplant Immunology*, *12*(3–4), 359–365. 10.1016/j.trim.2003.12.010 15157927 10.1016/j.trim.2003.12.010

[19] Smadja, D. M., Saubaméa, B., Susen, S., Kindo, M., Bruneval, P., Van Belle, E., Carpentier, A., et al. (2017). Bioprosthetic total artificial heart induces a profile of acquired hemocompatibility with membranes recellularization. *Journal of the American College of Cardiology,**70*(3), 404–406. 10.1016/j.jacc.2017.05.021 28705324 10.1016/j.jacc.2017.05.021

[20] Guyonnet, L., Detriché, G., Gendron, N., Philippe, A., Latremouille, C., Soret, L., & Smadja, D. M. (2021). Elevated Circulating stem cells level is observed one month after implantation of carmat bioprosthetic total artificial heart. *Stem Cell Reviews and Reports*, *17*(6), 2332–2337. 10.1007/s12015-021-10270-3 34622384 10.1007/s12015-021-10270-3

[21] Cheng, C. K., Wang, N., Wang, L., & Huang, Y. (2025). Biophysical and biochemical roles of shear stress on endothelium: A revisit and new insights. *Circulation Research*, *136*(7), 752–772. 10.1161/CIRCRESAHA.124.325685 40146803 10.1161/CIRCRESAHA.124.325685 PMC11949231

[22] Santos, F., Sum, H., Yan, D. C. L., & Brewer, A. C. (2025). Metaboloepigenetics: Role in the regulation of Flow-Mediated endothelial (Dys)Function and atherosclerosis. *Cells*, *14*(5), 378. 10.3390/cells14050378 40072106 10.3390/cells14050378 PMC11898952

[23] Kim, J.-E., Jeong, G.-J., Yoo, Y. M., Bhang, S. H., Kim, J. H., Shin, Y. M., … Yoon,J.-K. (2025). 3D bioprinting technology for modeling vascular diseases and its application. *Biofabrication*, 17(2). 10.1088/1758-5090/adc03a 10.1088/1758-5090/adc03a 40081017

[24] Capalbo, S., Polyakova, A., El Imane, Z., Khan, I., Kawai, T., Shindo, S., & Salinas, M. (2025). A comprehensive review of contemporary bioreactors for vascular inflammation studies. *Inflammation*. 10.1007/s10753-024-02231-y 10.1007/s10753-024-02231-y 39903422

[25] Blazeski, A., Floryan, M. A., Zhang, Y., Fajardo Ramírez, O. R., Meibalan, E., Ortiz-Urbina, J., & García-Cardeña, G. (2024). Engineering microvascular networks using a KLF2 reporter to probe flow-dependent endothelial cell function. *Biomaterials,**311*, 122686. 10.1016/j.biomaterials.2024.12268638971122 10.1016/j.biomaterials.2024.122686

[26] Marrone, G., Maeso-Díaz, R., García-Cardena, G., Abraldes, J. G., García-Pagán, J. C., Bosch, J., & Gracia-Sancho, J. (2015). KLF2 exerts antifibrotic and vasoprotective effects in cirrhotic rat livers: Behind the molecular mechanisms of Statins. *Gut*, *64*(9), 1434–1443. 10.1136/gutjnl-2014-308338 25500203 10.1136/gutjnl-2014-308338

[27] Marrone, G., Russo, L., Rosado, E., Hide, D., García-Cardeña, G., García-Pagán, J. C., & Gracia-Sancho, J. (2013). The transcription factor KLF2 mediates hepatic endothelial protection and paracrine endothelial-stellate cell deactivation induced by Statins. *Journal of Hepatology*, *58*(1), 98–103. 10.1016/j.jhep.2012.08.026 22989565 10.1016/j.jhep.2012.08.026

[28] Gracia-Sancho, J., Russo, L., García-Calderó, H., García-Pagán, J. C., García-Cardeña, G., & Bosch, J. (2011). Endothelial expression of transcription factor Kruppel-like factor 2 and its vasoprotective target genes in the normal and cirrhotic rat liver. *Gut*, *60*(4), 517–524. 10.1136/gut.2010.220913 21112949 10.1136/gut.2010.220913

[29] Heckel, E., Boselli, F., Roth, S., Krudewig, A., Belting, H. G., Charvin, G., & Vermot, J. (2015). Oscillatory flow modulates mechanosensitive klf2a expression through trpv4 and trpp2 during heart valve development. *Current Biology: CB*, *25*(10), 1354–1361. 10.1016/j.cub.2015.03.038 25959969 10.1016/j.cub.2015.03.038

[30] Gracia-Sancho, J., Villarreal, G., Zhang, Y., Yu, J. X., Liu, Y., Tullius, S. G., & García-Cardeña, G. (2010). Flow cessation triggers endothelial dysfunction during organ cold storage conditions: Strategies for Pharmacologic intervention. *Transplantation*, *90*(2), 142–149. 10.1097/TP.0b013e3181e228db 20606606 10.1097/TP.0b013e3181e228db PMC4522158

[31] Dar, W. A., Sullivan, E., Bynon, J. S., Eltzschig, H., & Ju, C. (2019). Ischaemia reperfusion injury in liver transplantation: Cellular and molecular mechanisms. *Liver International: Official Journal of the International Association for the Study of the Liver*, *39*(5), 788–801. 10.1111/liv.14091 30843314 10.1111/liv.14091 PMC6483869

[32] Oberhuber, R., Riede, G., Cardini, B., Bernhard, D., Messner, B., Watschinger, K., Maglione, M., et al. (2016). Impaired endothelial nitric oxide synthase homodimer formation triggers development of transplant vasculopathy - insights from a murine aortic transplantation model. *Scientific Reports,**6*, 37917. 10.1038/srep37917 27883078 10.1038/srep37917 PMC5121662

[33] Dabravolski, S. A., Sukhorukov, V. N., Kalmykov, V. A., Grechko, A. V., Shakhpazyan, N. K., & Orekhov, A. N. (2022). The role of KLF2 in the regulation of atherosclerosis development and potential use of KLF2-Targeted therapy. *Biomedicines*, *10*(2), 254. 10.3390/biomedicines10020254 35203463 10.3390/biomedicines10020254 PMC8869605

[34] Geelhoed, W. J., Moroni, L., & Rotmans, J. I. (2017). Utilizing the foreign body response to grow tissue engineered blood vessels in vivo. *Journal of Cardiovascular Translational Research*, *10*(2), 167–179. 10.1007/s12265-017-9731-7 28205013 10.1007/s12265-017-9731-7 PMC5437130

[35] Sparks, C. H. (1973). Silicone mandril method for growing reinforced autogenous Femoro-Popliteal artery grafts in situ. *Annals of Surgery*, *177*(3), 293–300. 10.1097/00000658-197303000-00009 4266308 10.1097/00000658-197303000-00009 PMC1355530

[36] Kutkut, I., Meens, M. J., McKee, T. A., Bochaton-Piallat, M. L., & Kwak, B. R. (2015). Lymphatic vessels: An emerging actor in atherosclerotic plaque development. *European Journal of Clinical Investigation*, *45*(1), 100–108. 10.1111/eci.12372 25388153 10.1111/eci.12372

[37] Wang, Y., Zhang, X., Li, X., Cheng, M., & Cui, X. (2025). The vascular microenvironment and its stem cells regulate vascular homeostasis. *Frontiers in Cell and Developmental Biology*, *13*, 1544129. 10.3389/fcell.2025.1544129 40114970 10.3389/fcell.2025.1544129 PMC11922910

[38] Ji, R. C. (2022). The role of lymphangiogenesis in cardiovascular diseases and heart transplantation. *Heart Failure Reviews*, *27*(5), 1837–1856. 10.1007/s10741-021-10188-5 34735673 10.1007/s10741-021-10188-5 PMC9388451

[39] Tremblay, P. L., Hudon, V., Berthod, F., Germain, L., & Auger, F. A. (2005). Inosculation of tissue-engineered capillaries with the host’s vasculature in a reconstructed skin transplanted on mice. *American Journal of Transplantation: Official Journal of the American Society of Transplantation and the American Society of Transplant Surgeons*, *5*(5), 1002–1010. 10.1111/j.1600-6143.2005.00790.x 15816880 10.1111/j.1600-6143.2005.00790.x

[40] Bačáková, L., Chlupáč, J., Filová, E., Musílková, J., Tomšů, J., Wu, Y. C., Brož, A., et al. (2024). Vascular damage and Repair - Are Small-Diameter vascular grafts still the holy Grail of tissue engineering? *Physiological Research,**73*(Suppl 1), S335–S363. 10.33549/physiolres.935294 38836460 10.33549/physiolres.935294 PMC11412351

[41] Moura, D., Pereira, A. T., Ferreira, H. P., Barrias, C. C., Magalhães, F. D., Bergmeister, H., & Gonçalves, I. C. (2023). Poly(2-hydroxyethyl methacrylate) hydrogels containing graphene-based materials for blood-contacting applications: From soft inert to strong degradable material. *Acta Biomaterialia*, *164*, 253–268. 10.1016/j.actbio.2023.04.031 37121371 10.1016/j.actbio.2023.04.031

[42] Fallon, M. E., Le, H. H., Bates, N. M., Yao, Y., Yim, E. K. F., Hinds, M. T., & Anderson, D. E. J. (2022). Hemocompatibility of micropatterned biomaterial surfaces is dependent on topographical feature size. *Frontiers in Physiology*, 13. 10.3389/fphys.2022.983187 10.3389/fphys.2022.983187 PMC9527343 36200053

[43] Yao, Y., Zaw, A. M., Anderson, D. E. J., Jeong, Y., Kunihiro, J., Hinds, M. T., & Yim, E. K. F (2023). Fucoidan and topography modification improved in situ endothelialization on acellular synthetic vascular grafts. *Bioactive Materials*, *22*, 535–550. 10.1016/j.bioactmat.2022.10.011 36330164 10.1016/j.bioactmat.2022.10.011 PMC9619221

[44] Tu, C., Zhang, Y., Xiao, Y., Xing, Y., Jiao, Y., Geng, X., & Feng, Z. (2022). Hydrogel-complexed small-diameter vascular graft loaded with tissue-specific vascular extracellular matrix components used for tissue engineering. *Biomaterials Advances*, *142*, 213138. 10.1016/j.bioadv.2022.213138 36219919 10.1016/j.bioadv.2022.213138

[45] Jin, Q., Yu, C., Xu, L., Zhang, G., Ju, J., & Hou, R. (2023). Combined light-cured and sacrificial hydrogels for fabrication of small-diameter bionic vessels by 3D Bioprinting. *Technology and Health Care*, *31*(4), 1203–1213. 10.3233/thc-220393 36872804 10.3233/THC-220393

[46] Filova, E., Steinerova, M., Travnickova, M., Knitlova, J., Musilkova, J., Eckhardt, A., Bacakova, L., et al. (2021). Accelerated in vitro recellularization of decellularized Porcine pericardium for cardiovascular grafts. *Biomedical Materials (Bristol England),**16*(2), 025024. 10.1088/1748-605X/abbdbd 33629665 10.1088/1748-605X/abbdbd

[47] Wilcox, E. C., & Edelman, E. R. (2022). Substratum interactions determine immune response to allogeneic transplants of endothelial cells. *Frontiers in Immunology*, *13*, 946794. 10.3389/fimmu.2022.946794 36003373 10.3389/fimmu.2022.946794 PMC9393654

[48] Smadja, D. M., Berkane, Y., Bentounes, N. K., Rancic, J., Cras, A., Pinault, C., Jeljeli, M., et al. (2025). Immune-privileged cord blood-derived endothelial colony-forming cells: Advancing immunomodulation and vascular regeneration. *Angiogenesis,**28*(2), 19. 10.1007/s10456-025-09973-9 40047974 10.1007/s10456-025-09973-9 PMC11885380

[49] Amersfoort, J., Eelen, G., & Carmeliet, P. (2022). Immunomodulation by endothelial cells — partnering up with the immune system? *Nature Reviews Immunology*, *22*(9), 576–588. 10.1038/s41577-022-00694-4 35288707 10.1038/s41577-022-00694-4 PMC8920067

[50] Vandenberghe, P., Delabie, J., de Boer, M., De Wolf-Peeters, C., & Ceuppens, J. L. (1993). In situ expression of B7/BB1 on Antigen-presenting cells And activated B cells: An immunohistochemical study. *International Immunology*, *5*(3), 317–321. 10.1093/intimm/5.3.317 7682106 10.1093/intimm/5.3.317

[51] Chen, M. B., Yang, A. C., Yousef, H., Lee, D., Chen, W., Schaum, N., Wyss-Coray, T., et al. (2020). Brain endothelial cells are exquisite sensors of age-related circulatory cues. *Cell Reports,**30*(13), 4418–4432.e4. 10.1016/j.celrep.2020.03.012 32234477 10.1016/j.celrep.2020.03.012 PMC7292569

[52] Pintavorn, P., & Ballermann, B. J. (1997). TGF-beta and the endothelium during immune injury. *Kidney International*, *51*(5), 1401–1412. 10.1038/ki.1997.192 9150451 10.1038/ki.1997.192

[53] Kotamraju, S., Matalon, S., Matsunaga, T., Shang, T., Hickman-Davis, J. M., & Kalyanaraman, B. (2006). Upregulation of immunoproteasomes by nitric oxide: Potential antioxidative mechanism in endothelial cells. *Free Radical Biology & Medicine*, *40*(6), 1034–1044. 10.1016/j.freeradbiomed.2005.10.052 16540399 10.1016/j.freeradbiomed.2005.10.052

[54] Tousoulis, D., Kampoli, A. M., Tentolouris, C., Papageorgiou, N., & Stefanadis, C. (2012). The role of nitric oxide on endothelial function. *Current Vascular Pharmacology*, *10*(1), 4–18. 10.2174/157016112798829760 22112350 10.2174/157016112798829760

[55] Goveia, J., Rohlenova, K., Taverna, F., Treps, L., Conradi, L.-C., Pircher, A., Carmeliet, P., et al. (2020). An integrated gene expression landscape profiling approach to identify lung tumor endothelial cell heterogeneity and angiogenic candidates. *Cancer Cell,**37*(1), 21–36.e13. 10.1016/j.ccell.2019.12.001 31935371 10.1016/j.ccell.2019.12.001

[56] Kreisel, D., Richardson, S. B., Li, W., Lin, X., Kornfeld, C. G., Sugimoto, S., Krupnick, A. S., et al. (2010). Cutting edge: MHC class II expression by pulmonary nonhematopoietic cells plays a critical role in controlling local inflammatory responses. *Journal of Immunology,**185*(7), 3809–3813. 10.4049/jimmunol.1000971 10.4049/jimmunol.1000971 PMC3897247 20810992

[57] Travaglini, K. J., Nabhan, A. N., Penland, L., Sinha, R., Gillich, A., Sit, R. V., Krasnow, M. A., et al. (2020). A molecular cell atlas of the human lung from single-cell RNA sequencing. *Nature,**587*(7835), 619–625. 10.1038/s41586-020-2922-4 33208946 10.1038/s41586-020-2922-4 PMC7704697

[58] Dauphinee, S. M., & Karsan, A. (2006). Lipopolysaccharide signaling in endothelial cells. Laboratory investigation; a. *Journal of Technical Methods and Pathology*, *86*(1), 9–22. 10.1038/labinvest.3700366 10.1038/labinvest.3700366 16357866

[59] Liao, J. K. (2013). Linking endothelial dysfunction with endothelial cell activation. *The Journal of Clinical Investigation*, *123*(2), 540–541. 10.1172/JCI66843 23485580 10.1172/JCI66843 PMC3561809

[60] Ghaleb, A. M., & Yang, V. W. (2017). Krüppel-like factor 4 (KLF4): What we currently know. *Gene*, *611*, 27–37. 10.1016/j.gene.2017.02.025 28237823 10.1016/j.gene.2017.02.025 PMC5391259

[61] Shen, B., Smith, R. S., Hsu, Y. T., Chao, L., & Chao, J. (2009). Kruppel-like factor 4 is a novel mediator of Kallistatin in inhibiting endothelial inflammation via increased endothelial nitric-oxide synthase expression. *The Journal of Biological Chemistry*, *284*(51), 35471–35478. 10.1074/jbc.M109.046813 19858207 10.1074/jbc.M109.046813 PMC2790976

[62] SenBanerjee, S., Lin, Z., Atkins, G. B., Greif, D. M., Rao, R. M., Kumar, A., Jain, M. K., et al. (2004). KLF2 is a novel transcriptional regulator of endothelial proinflammatory activation. *The Journal of Experimental Medicine,**199*(10), 1305–1315. 10.1084/jem.20031132 15136591 10.1084/jem.20031132 PMC2211816

[63] Weber, J., Weber, M., Feile, A., Schlensak, C., & Avci-Adali, M. (2023). Development of an in vitro blood vessel model using autologous endothelial cells generated from Footprint-Free HiPSCs to analyze interactions of the endothelium with blood cell components and vascular implants. *Cells*, *12*(9), 1217. 10.3390/cells12091217 37174617 10.3390/cells12091217 PMC10177426

[64] Ma, Y., Zhang, Z., Chen, R., Shi, R., Zeng, P., Chen, R., F Chen, A., et al. (2019). NRP1 regulates HMGB1 in vascular endothelial cells under high homocysteine condition. *American Journal of Physiology Heart and Circulatory Physiology,**316*(5), H1039–H1046. 10.1152/ajpheart.00746.2018 30767669 10.1152/ajpheart.00746.2018

[65] Cai, X., Biswas, I., Panicker, S. R., Giri, H., & Rezaie, A. R. (2019). Activated protein C inhibits lipopolysaccharide-mediated acetylation and secretion of high-mobility group box 1 in endothelial cells. *Journal of Thrombosis and Haemostasis: JTH*, *17*(5), 803–817. 10.1111/jth.14425 30865333 10.1111/jth.14425 PMC6494677

[66] Jia, H., Moore, M., Wadhwa, M., & Burns, C. (2024). Human iPSC–derived endothelial cells exhibit reduced immunogenicity in comparison with human primary endothelial cells. *Stem Cells International,**2024*, 6153235. 10.1155/sci/6153235 39687754 10.1155/sci/6153235 PMC11649354

[67] Deuse, T., Hu, X., Gravina, A., Wang, D., Tediashvili, G., De, C., & Schrepfer, S. (2019). Hypoimmunogenic derivatives of induced pluripotent stem cells evade immune rejection in fully immunocompetent allogeneic recipients. *Nature Biotechnology*, *37*(3), 252–258. 10.1038/s41587-019-0016-3 30778232 10.1038/s41587-019-0016-3 PMC6419516

[68] Meyer, S. R., Nagendran, J., Desai, L. S., Rayat, G. R., Churchill, T. A., Anderson, C. C., & Ross, D. B. (2005). Decellularization reduces the immune response to aortic valve allografts in the rat. *The Journal of Thoracic and Cardiovascular Surgery*, *130*(2), 469–476. 10.1016/j.jtcvs.2005.03.021 16077415 10.1016/j.jtcvs.2005.03.021

[69] Antonopoulos, C. N., Papakonstantinou, N. A., Hardy, D., & Lyden, S. P. (2019). Editor’s Choice – Cryopreserved allografts for arterial reconstruction after Aorto-Iliac infection: A systematic review and Meta-Analysis. *European Journal of Vascular and Endovascular Surgery*, *58*(1), 120–128. 10.1016/j.ejvs.2019.03.003 31202580 10.1016/j.ejvs.2019.03.003

[70] Doty, J. R., Salazar, J. D., Liddicoat, J. R., Flores, J. H., & Doty, D. B. (1998). Aortic valve replacement with cryopreserved aortic allograft: Ten-Year experience. *The Journal of Thoracic and Cardiovascular Surgery*, *115*(2), 371–380. 10.1016/S0022-5223(98)70281-8 9475532 10.1016/S0022-5223(98)70281-8

[71] Heo, S. H., Kim, Y. W., Woo, S. Y., Park, Y. J., Kim, D. K., & Chung, D. R. (2017). Recent results of in situ abdominal aortic reconstruction with cryopreserved arterial allograft. *European Journal of Vascular and Endovascular Surgery*, *53*(2), 158–167. 10.1016/j.ejvs.2016.07.090 27592735 10.1016/j.ejvs.2016.07.090

[72] Minga Lowampa, E., Holemans, C., Stiennon, L., Van Damme, H., & Defraigne, J. O. (2016). Late fate of cryopreserved arterial allografts. *European Journal of Vascular and Endovascular Surgery*, *52*(5), 696–702. 10.1016/j.ejvs.2016.08.005 27614553 10.1016/j.ejvs.2016.08.005

[73] Berkane, Y., Oubari, H., van Dieren, L., Charlès, L., Lupon, E., McCarthy, M., & Lellouch, A. G. (2024). Tissue engineering strategies for breast reconstruction: A literature review of current advances and future directions. *Annals of Translational Medicine*, *12*(1), 15. 10.21037/atm-23-1724 38304901 10.21037/atm-23-1724 PMC10777243

[74] Majid, Q. A., Ghimire, B. R., Merkely, B., Randi, A. M., Harding, S. E., Talman, V., & Földes, G. (2024). Generation and characterisation of scalable and stable human pluripotent stem cell-derived microvascular-like endothelial cells for cardiac applications. *Angiogenesis*. 10.1007/s10456-024-09929-5 38775849 10.1007/s10456-024-09929-5 PMC11303486

[75] Luo, A. C., Wang, J., Wang, K., Zhu, Y., Gong, L., Lee, U., & Melero-Martin, J. M. (2024). A streamlined method to generate endothelial cells from human pluripotent stem cells via transient doxycycline-inducible ETV2 activation. *Angiogenesis*. 10.1007/s10456-024-09937-5 38969874 10.1007/s10456-024-09937-5 PMC11577265

[76] Smadja, D. M. (2019). Vasculogenic stem and progenitor cells in human: Future cell therapy product or liquid biopsy for vascular disease. *Advances in Experimental Medicine and Biology*, *1201*, 215–237. 10.1007/978-3-030-31206-0_11 31898789 10.1007/978-3-030-31206-0_11

[77] Smadja, D. M., Melero-Martin, J. M., Eikenboom, J., Bowman, M., Sabatier, F., & Randi, A. M. (2019). Standardization of methods to quantify and culture endothelial colony-forming cells derived from peripheral blood: Position paper from the international society on thrombosis and haemostasis SSC. *Journal of Thrombosis and Haemostasis: JTH*, *17*(7), 1190–1194. 10.1111/jth.14462 31119878 10.1111/jth.14462 PMC7028216

[78] Blandinieres, A., Randi, A. M., Paschalaki, K. E., Guerin, C. L., Melero-Martin, J. M., & Smadja, D. M. (2023). Results of an international survey about methods used to isolate human endothelial colony-forming cells (ECFCs): Guidance from the scientific and standardization committee on vascular biology of the international society of thrombosis and hemostasis. *Journal of thrombosis and haemostasis: JTH,**S1538–7836*(23), 00498–1. 10.1016/j.jtha.2023.06.014 10.1016/j.jtha.2023.06.014 37336438

[79] Smadja, D. M., Mauge, L., Rancic, J., Gaussem, P., Feraud, O., Oudrhiri, N., & Bennaceur-Griscelli, A. (2024). Comparative evaluation of endothelial Colony-Forming cells from cord and adult blood vs. Human embryonic stem Cell-Derived endothelial cells: Insights into therapeutic angiogenesis potential. *Stem Cell Reviews and Reports*. 10.1007/s12015-024-10830-3 39612122 10.1007/s12015-024-10830-3

[80] Wu, K. X., Yeo, N. J. Y., Ng, C. Y., Chioh, F. W. J., Fan, Q., Tian, X., Cheung, C., et al. (2022). Hyaluronidase-1-mediated glycocalyx impairment underlies endothelial abnormalities in polypoidal choroidal vasculopathy. *BMC biology,**20*(1), 47. 10.1186/s12915-022-01244-z 35164755 10.1186/s12915-022-01244-z PMC8845246

[81] Wei, P., Milbauer, L. C., Enenstein, J., Nguyen, J., Pan, W., & Hebbel, R. P. (2011). Differential endothelial cell gene expression by African Americans versus Caucasian americans: A possible contribution to health disparity in vascular disease and cancer. *BMC Medicine*, *9*, 2. 10.1186/1741-7015-9-2 21223544 10.1186/1741-7015-9-2 PMC3029215

[82] Berkane, Y., Kostyra, D. M., Chrelias, T., Randolph, M. A., Lellouch, A. G., Cetrulo, C. L., Duisit, J., et al. (2023). The autonomization principle in vascularized flaps: An alternative strategy for composite tissue scaffold in vivo revascularization. *Bioengineering (Basel, Switzerland),**10*(12), 1440. 10.3390/bioengineering10121440 38136031 10.3390/bioengineering10121440 PMC10740989

[83] Kulik, A., Ruel, M., Jneid, H., Ferguson, T. B., Hiratzka, L. F., Ikonomidis, J. S., Zimmerman, L., et al. (2015). Secondary prevention after coronary artery bypass graft surgery: A scientific statement from the American Heart Association. *Circulation,**131*(10), 927–964. 10.1161/cir.0000000000000182 25679302 10.1161/CIR.0000000000000182

[84] Ladak, S. S., McQueen, L. W., Layton, G. R., Aujla, H., Adebayo, A., & Zakkar, M. (2022). The role of endothelial cells in the onset, development and modulation of vein graft disease. *Cells*, *11*(19), 3066. 10.3390/cells11193066 36231026 10.3390/cells11193066 PMC9561968

[85] Yan, H., Cheng, C., Song, Y., Luo, L., Liu, D., & Zhang, D. (2025). Revealing the process of vein graft failure: A panoramic review from etiology analysis to mechanism explanation and treatment strategy. *Cardiovascular Drugs and Therapy*. 10.1007/s10557-025-07711-3 40439962 10.1007/s10557-025-07711-3

[86] Comerota, A. J. (2001). Endovascular and surgical revascularization for patients with intermittent claudication. *The American Journal of Cardiology*, *87*(12), 34–43. 10.1016/s0002-9149(01)01674-5 11434898 10.1016/s0002-9149(01)01674-5

